# Sign Language Motion Generation from Sign Characteristics

**DOI:** 10.3390/s23239365

**Published:** 2023-11-23

**Authors:** Manuel Gil-Martín, María Villa-Monedero, Andrzej Pomirski, Daniel Sáez-Trigueros, Rubén San-Segundo

**Affiliations:** 1 Grupo de Tecnología del Habla y Aprendizaje Automático (T.H.A.U. Group), Information Processing and Telecommunications Center, Escuela Técnica Superior de Ingenieros de Telecomunicación, Universidad Politécnica de Madrid, 28040 Madrid, Spain; maria.villa.monedero@alumnos.upm.es; 2 Alexa AI, Aleja Grunwaldzka 472, 80-309 Gdańsk, Poland; pomirsa@amazon.com; 3 Alexa AI, C. de Ramírez de Prado 5, 28045 Madrid, Spain

**Keywords:** motion generation, motion dataset, sign language, sign phonemes, HamNoSys, landmarks extraction, interpolation, padding strategies

## Abstract

This paper proposes, analyzes, and evaluates a deep learning architecture based on transformers for generating sign language motion from sign phonemes (represented using HamNoSys: a notation system developed at the University of Hamburg). The sign phonemes provide information about sign characteristics like hand configuration, localization, or movements. The use of sign phonemes is crucial for generating sign motion with a high level of details (including finger extensions and flexions). The transformer-based approach also includes a stop detection module for predicting the end of the generation process. Both aspects, motion generation and stop detection, are evaluated in detail. For motion generation, the dynamic time warping distance is used to compute the similarity between two landmarks sequences (ground truth and generated). The stop detection module is evaluated considering detection accuracy and ROC (receiver operating characteristic) curves. The paper proposes and evaluates several strategies to obtain the system configuration with the best performance. These strategies include different padding strategies, interpolation approaches, and data augmentation techniques. The best configuration of a fully automatic system obtains an average DTW distance per frame of 0.1057 and an area under the ROC curve (AUC) higher than 0.94.

## 1. Introduction

Motion generation is an innovative research field which consists of producing movements, gestures, or animations by computer systems. This process involves the use of mathematical algorithms to create dynamic, natural, and fluid motions that simulate human movements, enhancing human–computer interactions. Motion generation has many different applications, such as movement of virtual characters in video games or animated films, robotics, and sign language representation in communication systems for deaf people. In sign language communication systems, motion generation allows for an increase in the naturality of interactive avatars or virtual assistants that respond with a sign language output. Increasing naturality allows for the development of friendly communication systems between deaf and hearing people. These systems empower and enhance the communication capabilities of deaf individuals, encouraging inclusivity and facilitating their integration into various social and professional scenarios.

Most state-of-the-art sign language motion generation systems are based on expert rules or prerecorded movements. This work proposes to train a module to automatically generate sign language motion from sign phonemes, represented using HamNoSys [1]. The proposed generation system is based on deep learning using a transformer-based approach [2]. HamNoSys is a phonetic transcription system for sign language, which includes sign characteristics or phonemes such as hand location, shape, and movement.

To the best of the authors’ knowledge, the proposed system is the first motion generation system for sign language based on transformers. The main contributions of this paper are the following:

- Proposal and evaluation of a deep learning architecture based on transformers for generating the sequence of landmarks to represent a sign.
- This proposed approach also includes a stop module for deciding the end of the generation process. This stop module is also evaluated in different scenarios.
- Additional analyses for improving the system accuracy, considering different padding strategies, interpolation approaches, and data augmentation techniques.

This paper is organized as follows. Section 2 reviews the literature, discussing different previous studies related to the topic of this work. Section 3 describes the dataset used for this work. Section 4 contains the methods used, including the system architecture, specifying the motion generation, the stop detection module, and the strategies to improve the system performance. Section 5 includes the evaluation details and discusses the experiments and the obtained results, and Section 6 presents the results, combining motion generation and stop detection in the same system. Finally, Section 7 summarizes the main conclusions of the paper.

## 2. Related Work

Motion generation is a research field aimed at developing realistic 2D or 3D animations based on different inputs such as image sequences, speech recordings, or text descriptions. In the literature, we found several datasets for motion generation and different evaluation metrics to assess the quality of generated motion. This section reviews some previous works about motion generation, including information about evaluation metrics and specific works related to sign language.

### 2.1. Motion Generation

Within human motion modeling [3,4,5,6], motion generation aims at creating realistic animations to simulate human gestures and movements in virtual avatars. This generation can be achieved using different types of information as inputs to the systems: image sequences, speech recordings, or text descriptions that represent a specific movement. Some previous works [7,8] focused on generating articulated motion RGB video sequences by estimating the human pose based on a single frame image. The proposed approach incorporated constraints from 2D/3D skeleton points and used generative adversarial networks (GAN) to generate a more human-like 3D human model. Other works have focused on generating animated avatars from audio. For instance, Shlizerman et al. [9] developed an avatar that moved its hands similarly to how a pianist or violinist would do using music as input. The authors trained a long short-term memory (LSTM) network considering audio features such as mel-frequency cepstral coefficients (MFCC) from violin and piano recital videos. Regarding the use of text description to generate motion, a previous work [10] generated 3D human motion sequences given a prescribed action type. The proposed approach used a conditional temporal variational autoencoder (VAE) approach endowed with Lie algebra representation.

### 2.2. Metrics for Motion Generation

Regarding the assessment of the generated motion, there are several possible ways to proceed. Some metrics, like smoothness, refer to how natural the generated motion is, and others, such as diversity or multimodality [10], refer to how realistic the generated motion is, including variance across all action categories or variety within each action class. However, there exist metrics that objectively determine how similar the generated motion is compared to the ground truth. Some of these objective metrics are Fréchet inception distance (FID), probability of correct key points (PCK), average position error (APE), and dynamic time warping (DTW). FID refers to the distance between the feature distribution of generated motion and the ground truth [10]. PCK scores evaluate the probability of pose key points to be close to the ground truth key points up to a specific threshold, with higher PCK corresponding to better pose generations [11]. APE covers the error for all the locations of the human pose (landmarks) at all times [12], computing the average position error. DTW finds an optimal alignment between two landmark time series by non-linearly warping them. A lower DTW corresponds to better landmark sequence generations [11]. 

Even though all these metrics have been used in the literature, some previous works have highlighted the efficacy of DTW compared to others, especially for aligning landmarks sequences. For example, a previous work [13], focusing on the motion capture data of the human gait, used DTW as a measure to align time instants, concluding that DTW is an effective technique in motion data analysis. In addition, another previous work [14] used a DTW-based metric to assess Kinect-enabled home-based physical rehabilitation exercises. They used this metric to compute motion similarity between two time series from an individual user and a virtual coach. They concluded that a DTW-based metric could be effectively used for the automatic performance evaluation of motion exercises. Moreover, a recent work [15] focused on determining which is the best automated metric for text to motion generation. The authors concluded that commonly used metrics such as R-Precision (a distance-based metric that measures the rate of correct motion prompt pair matchings) show strong correlations with the quality of the generated movement. 

In this work, we use a DTW-based metric to evaluate the proposed landmark generation model. The metric consists of using DTW to align the sequences of ground truth and generated landmarks, and computing the average error between them.

### 2.3. Sign Language Generation

Some previous works in the literature have directly focused on sign language motion generation, exploring the interplay between linguistic expression through videos or textual descriptions and physical movements that represent that language.

A recent work [16] proposed a pose-based approach for gloss prediction using a transformer model and datasets of Word-Level American Sign Language (WLASL) [17]. Particularly, they used WLASL100, which contains 2038 videos from 100 glosses and 97 signers, and WLASL300, which includes 5117 videos from 300 glosses and 109 signers. The authors achieved the top 1% recognition accuracy of 80.9% in WLASL100 and 64.21% in WLASL300 via a key frame extraction technique that used histogram difference and Euclidean distance metrics to select and drop redundant frames. This work also used data augmentation based on joint angle rotation. Another work [18] extracted landmarks from videos of British Sign Language using MediaPipe, but in this work, the landmarks were used as input to CNN and LSTM-based classifiers.

Some works focused on generating motion from natural language. For example, in a previous work [12], the authors built a neural architecture able to learn a joint embedding of language and pose, mapping linguistic concepts and motion animations to the same representation space. They used the KIT Motion-Language Dataset, reaching PCK scores of 70% when using a threshold of 55 mm. Another work [19] used complex natural language sentences to generate motion through an encoder-decoder structure. They used a hierarchical two-stream model (pose and sentence encoders) along with a pose discriminator. In this case, the model also learned a joint embedding for both pose and language.

Other works generated motion from speech. For instance, a previous work [11] focused on converting speech segments into sign language through a cross-modal discriminator, allowing the network to correlate between poses and speech time-steps. They obtained a DTW distance of 14.05 over an Indian Sign Language dataset. Another work [20] used a BERT encoder to transform source sentences to context vectors and a transformer decoder to generate Korean Sign Language sequences.

Finally, a previous paper [21] provided a method to automate the process of Indian Sign Language generation using plain RGB images. The method leverages animation data, using an intermediate 2D OpenPose representation, to train a sign language generation model.

### 2.4. Sequence Generation Based on Transformers

The state-of-the-art technology for sequence generation is based on transformers. This technology has been used to generate different types of sequences. Li et al. [22] used a transformer-based architecture for speech synthesis. Their work focused on the use of a multi-head self-attention mechanism to enable end-to-end neural text-to-speech synthesis, similar to Tacotron2 [23]. By leveraging the power of multi-head and self-attention mechanisms, their work built the decoder and encoder in parallel, resulting in improved training efficiency. The transformer TTS model takes phoneme sequences as input and predicts mel-spectrogram sequences as output (these are converted to audio sequences with a vocoder).

This previous system [22] has a strong analogy with the system developed in this paper: instead of generating speech from phonemes, we target the generation of sign language motion from sign phonemes. To the best of the authors’ knowledge, in the literature, there is not any previous work focused on generating Spanish Sign Language (LSE: Lengua de Signos Española) using transformers (state of the art technology for modelling sequences). This paper proposes the first LSE generation system from sign phonemes or characteristics.

## 3. Dataset

**Figure 1:**
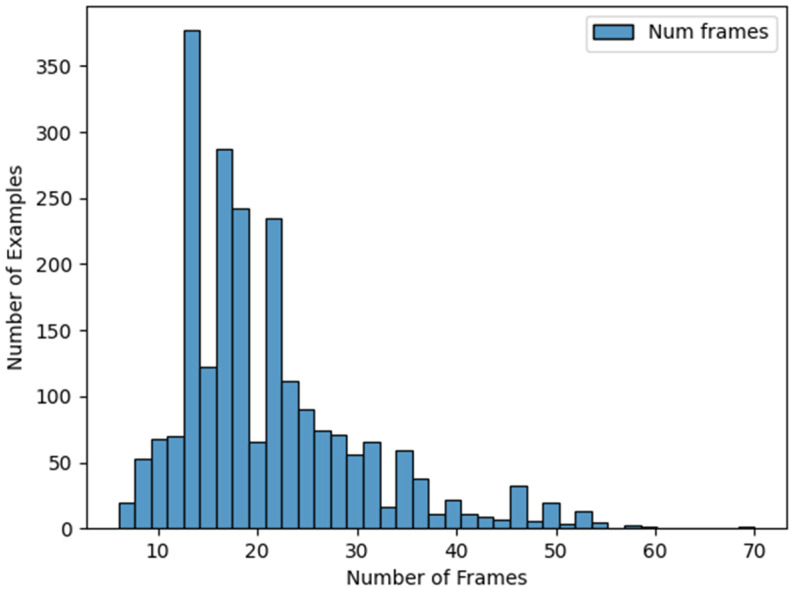
Histogram of frames per sign.

The dataset used in this work includes sign descriptions (phonemes specified in HamNoSys), sign videos, and landmarks for each frame of the videos [24]. The dataset includes 754 different signs represented by three different subjects with three different orientations. The synthetic videos were generated using the software developed by the Virtual Humans Group in the School of Computing Sciences at the University of East Anglia [25]. This software can be used on terms equivalent to Creative Commons BY-ND, allowing its use for research. This dataset includes content of a total of 6786 signs. For the experiments in this work, we used only the orientation facing forward: 2262 sign representations in total (754 signs represented by three different subjects). For each video generated we periodically obtained a frame, resulting in an average of 21 frames per sign. Figure 1 shows a histogram of frames per sign, where it is possible to observe that most of the signs contain between 10 and 30 frames.

### 3.1. HamNoSys or SiGML Sign Characteristics

**Figure 2:**
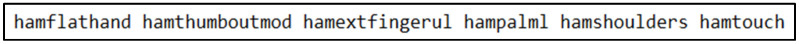
SiGML example representation of the gloss “AMO”.

This dataset includes sign phonemes represented using HamNoSys [1]. This notation system is transformed into Signing Gesture Markup Language (SiGML) annotations for a better processing [26,27]. SiGML is a simplified XML (Extensible Markup Language) format that preserves the same information as the HamNoSys description of a sign but is designed to be processed by computers. This notation includes aspects related to the body and specifically to the hand movements that describe the motion used to perform a sign. For example, Figure 2 includes the SiGML annotation for the gloss “AMO”, which means “love”. The sign characteristics or sign phonemes (coded using HamNoSys notation) are crucial in the context of sign motion generation because they univocally represent every sign, and they also contain a detailed description of the sign representation. This way, they are the best information we can introduce into the transformer architecture in order to generate the landmark sequences required to represent sign language.

### 3.2. Landmarks Extraction Using MediaPipe

This dataset includes landmarks (the x and y coordinates) of 57 pose and hand relevant points extracted with MediaPipe [28] for each frame. All coordinates have values in the [0.0, 1.0] interval considering the height and width of the input images. 

**Figure 3:**
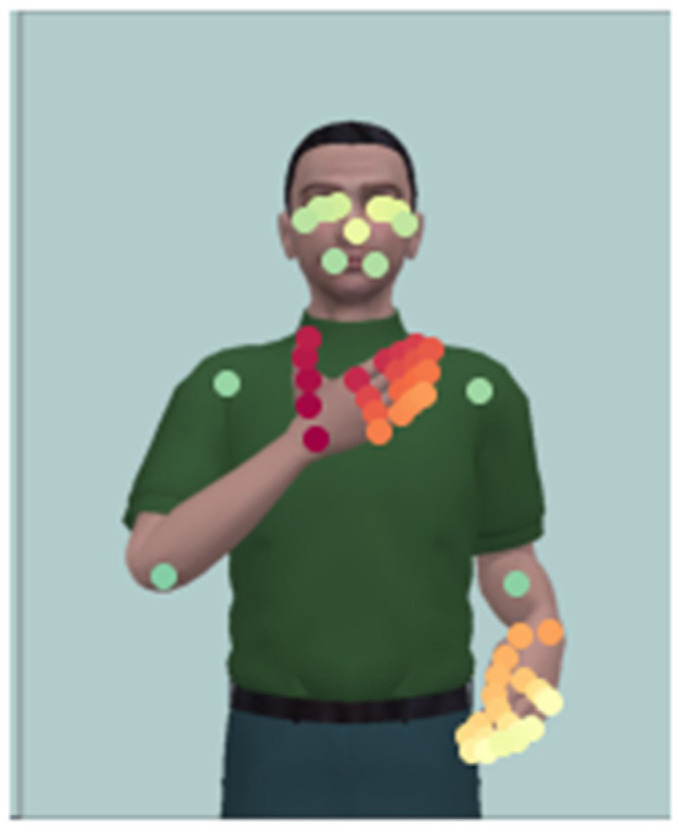
Landmarks over a frame from the sign “AMO” performed by one of the avatars (red landmarks are related to right hand, orange and yellow ones are related to the left hand, and green ones are related to the body).

These 57 landmarks specify 15 locations of the body including the face, shoulders, and elbows, and 21 locations for each hand including the wrist and four points along each of the five fingers. Figure 3 shows the extracted landmarks over a frame.

For this representation, it was necessary to combine MediaPipe Pose and MediaPipe Hands models to extract information from the whole body, and from both hands without ambiguity.

## 4. Methods

This section describes the methods used in this work: a transformer-based architecture to generate motion, which includes a stop detector. This description includes also the different variants considered in the experiments: several padding strategies, interpolation approaches, and data augmentation techniques.

### 4.1. System Architecture

In our proposal, we used a transformer-based architecture, similar to the architecture used in a previous work [22] for speech generation. In our case, we made certain modifications to adapt the inputs and outputs of the modules constituting the transformer (encoder and decoder modules): translating sign characteristics into landmark-based motion. The system includes a stop mechanism to limit the duration of the generated sequence.

**Figure 4:**
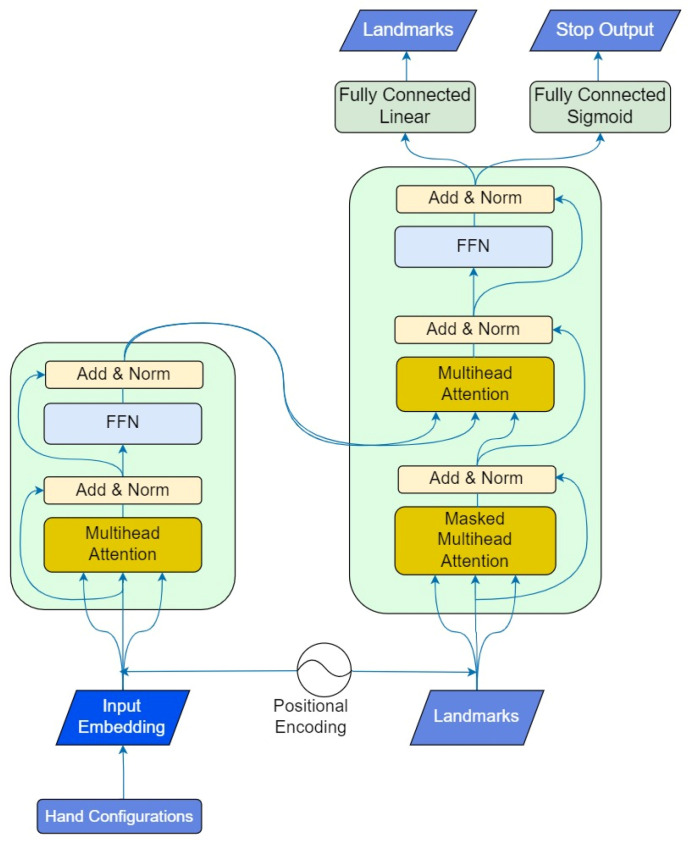
Diagram of the whole architecture to generate motion from the sign characteristics.

To develop this architecture, we used a transformer code implemented with keras_nlp [29]. In our architecture, the input data consist of the sign phonemes for the encoder, and the landmarks for the decoder. The encoder takes the sign phonemes and processes them using self-attention and feed-forward neural networks. Self-attention allows the model to weigh the different sign characteristics in the encoder input data, emphasizing the most relevant ones for each output. The decoder generates an output sequence of landmarks based on the sign phonemes and previously generated landmarks. The decoder output includes the predicted landmarks at this step, and also a binary output for indicating the end of the sign (stop token) (Figure 4). 

The encoder module codes the sign phonemes in order using a token and position embedding layer, which prepares the data for further processing by the decoder. This layer sums a token and position embedding to generate an embedding vector for each sign phoneme. The decoder takes a sequence of landmark vectors as inputs, which do not require tokenization, so only a positional embedding layer is needed. This layer learns a position embedding for decoder inputs sequences.

The decoder aims to predict the next set of landmarks based on the encoder information and the previous landmarks. 

In summary, the system generates two outputs: the predicted landmarks and a stop output, which indicates the end of the generated sequence. The system architecture incorporated two distinct loss functions to optimize its performance. The mean absolute error (MAE) was employed for the generation of landmarks, providing a measure of the absolute difference between the predicted and ground truth landmark coordinates. Simultaneously, the binary cross-entropy loss function was strategically integrated into the architecture for the binary classification of the stop detector. Various techniques for representing each sign through landmarks and designing the stop output will be analyzed in the following subsections.

To implement the whole system including the landmark generation and the stop detection layer, we have included two different output layers in the decoder (Figure 4). These layers are trained independently: the transformer is trained for landmark generation, and then only the stop layer is retrained considering the stop output. By using this approach, the transformer is capable of simultaneously predicting both the landmarks and the stop output. The end of the landmark sequence is determined by defining a threshold in the stop output: when the stop output is higher than the threshold, the end is detected. In the experiments, we will analyze the effect of this threshold.

The primary focus of the proposed deep learning architecture based on transformers is generating sign language motion sequences based on landmarks, using sign phonemes as input.

### 4.2. Motion Generation

This section outlines the processes undertaken for generating the landmarks using the transformer architecture. 

#### 4.2.1. Formatting the Data

To generate the predicted landmarks for the transformer, we used a subset of the database described in Section 3. 

**Figure 5:**
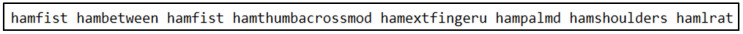
SiGML example representation of the letter “A”.

For the encoder’s inputs, we used the SiGML representations of the signs. We only used the sign phonemes corresponding to the manual aspects (hand characteristics: hand configurations, locations, and movements). These characteristics are compiled in a single line, as shown in Figure 5. 

**Figure 6:**
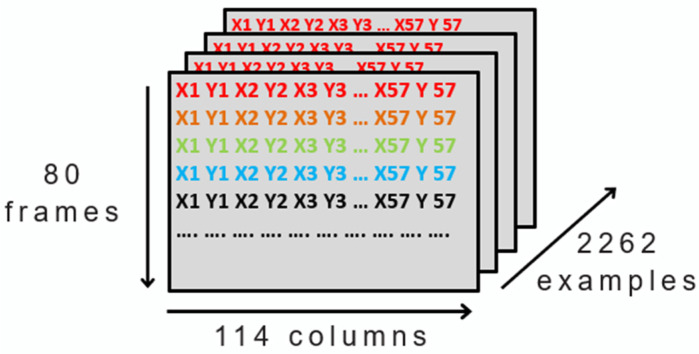
Representation of the decoder input format (different colors for each frame landmarks).

Secondly, for the decoder’s input we used the landmarks described in Section 3. Additionally, we fixed the number of frames per example to 80 because of the duration of the longest sign. By doing so, all examples had equal number of frames, enabling us to construct a three-dimensional matrix that includes all examples. This resulted in 2262 examples, with each sign consisting of 80 frames, and 57 landmarks (with two coordinates, x and y) that makes 114 numbers in total, as shown in Figure 6.

#### 4.2.2. Tokenizing the Encoder Inputs

In our system, only the encoder inputs need to be tokenized. To accomplish this, we considered the WordPiece tokenization algorithm, used by BERT and other models, that splits the text, in our case the hand configurations, into smaller units called tokens [30]. 

We trained the transformer considering two reserved tokens:

- [PAD]: Padding tokens which are appended to the input sequence length when the input sequence length is shorter than the maximum sequence length.
- [UNK]: Unknown tokens.

Here is an example of tokenizing and detokenizing an entry of the encoder, where next indicates the end of the sequence.

- Hand Configurations: hamflathand hamthumboutmod hamextfingeru hampalmu hamshoulders hamlrat hamparbegin hammovei hamarcu hamreplace hamextfingerui hampalmd hamparend hamrepeatfromstart next next
- Tokens: tf.Tensor ([49 43 38 47 41 40 34 86 90 46 131 44 35 55 36 36], shape = (16,), dtype = int32)
- Recovered text after detokenizing: hamflathand hamthumboutmod hamextfingeru hampalmu hamshoulders hamlrat hamparbegin hammovei hamarcu hamreplace hamextfingerui hampalmd hamparend hamrepeatfromstart next next

#### 4.2.3. Landmark Generation

Having defined the entire data processing pipeline, the last step is to construct the transformer model. The inputs and output of the transformer differ during training and inference:

- During training, the tokenized sign characteristics are passed as inputs to the encoder. The decoder inputs are the landmarks of the first frames [0 to end −1 frames] and the output are the last frames [1 to end frames]. This way, the model is autoregressive (the output of the current step depends on previous steps), being trained in teacher forcing mode (using previous ground truth landmarks for predicting the next landmark).
- During testing, the tokenized sign characteristics are also passed as inputs to the encoder. The main difference is in the decoder. The inputs of the decoder start with the landmarks of a resting position and, iteratively, the predicted landmarks generated by the transformer (next frame) are fed back as inputs to the decoder, allowing the transformer to predict the next set of landmarks based on the previous ones (autoregressive loop).

**Figure 7:**
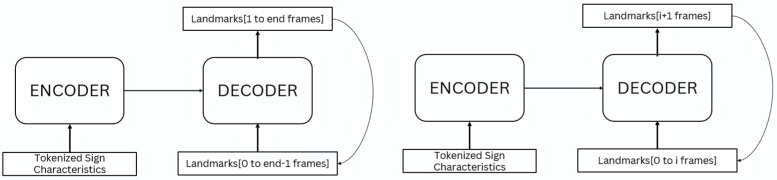
Block diagrams of the transformer inputs.

Figure 7 shows two simplified block diagrams of the training and testing processes, respectively. 

### 4.3. Stop Detection

**Figure 8:**
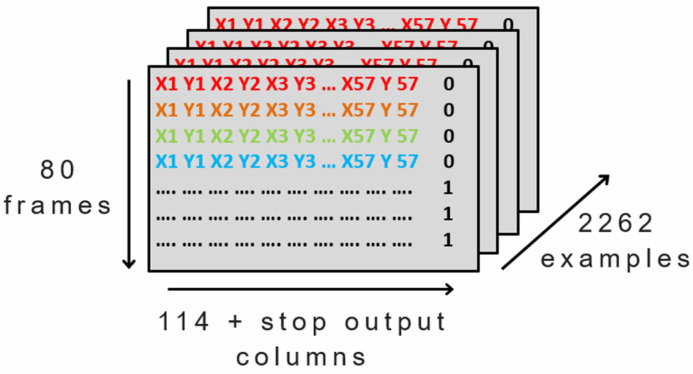
Representation of the decoder input with the stop information.

The stop information (indicating with 1 when to stop) is incorporated into every frame, as an additional column: 0 when a sign is taking place, and 1 to represent the end of a sign (Figure 8).

### 4.4. Padding Strategies

In this work, we implemented and evaluated several padding strategies for making the sign language sequences have the same length.

In the first strategy, zero padding, all the signs of the landmark-based sequences are padded with zeros at the end until they reach a fixed maximum length (80 frames in our case). This approach extends the shorter sequences to match the length of the longest sequence in the dataset. The second strategy, sign padding, consists of repeating the sign until the sequence reaches a fixed maximum length. Finally, the third strategy, hybrid padding, combines both ideas, padding with zeros and repetition of the sign: an intercalation of several zero frames is included before repeating the sign. In this work, three zero frames have been included between sign sequences.

These padding strategies only affect the landmarks. The stop information is fixed to 0 for the first occurrence of the sign and 1 for the rest of the frames until the maximum length is reached.

**Figure 9:**
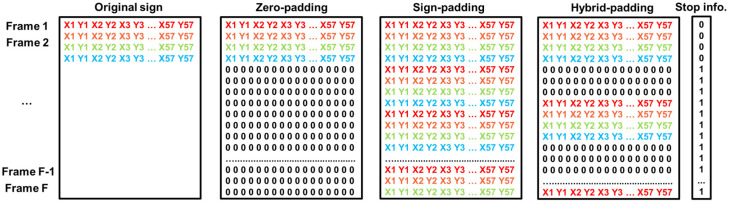
Representation of the different padding strategies considered in this work. The stop information is the same in all cases.

Figure 9 shows an example of the three padding strategies over a hypothetical original sign with four frames and considering F as the maximum length (80 in this work). As observed in the figure, the stop column is equal independently of the padding strategy: it has zeros for the actual frames of the sign (the first time that it appears) and ones for the rest of the frames until the maximum length of the sign is reached.

In the experiment section, the impact of the padding strategy will be evaluated in both transformer outputs: landmark generation and stop detection.

### 4.5. Frame Interpolation

The proposed approach based on a transformer predicts the landmarks of a frame given in the previous frames, generating the whole sequence in an iterative process. This prediction is more difficult when there are big differences in consecutive frames. One interesting idea to facilitate the learning process of the transformer is to reduce these differences by interpolating intermediate frames. We considered and evaluated a linear interpolation of one, two, or three intermediate frames between two original frames.

### 4.6. Data Augmentation

Data augmentation is a technique commonly used in machine learning to artificially increase the size and diversity of the training subset by applying different transformations to the existing data. This helps to improve the generalization and robustness of machine learning models. In the context of landmark representations, we applied two data augmentation techniques: shifting and zooming.

For shifting, we added a random number between −0.2 and 0.2 to all the landmark coordinates in a sign, displacing the signing subject.

For zooming, we compressed or expanded the images by 10% from the center of the scene. First, the center was subtracted from the landmark points, and then we multiplied each coordinate of the landmark points by a random number between 0.9 and 1.1. And at the end, we summed the center to the landmarks. This process uniformly compresses or expands the size of the landmark representation from the center of the scene.

These techniques increased the diversity of data, which could enable the model to better handle different positions, sizes, and scales of landmarks, improving its ability to accurately identify and localize landmarks in unseen and diverse data. We evaluated the impact of introducing these additional data during the training process.

## 5. Experiments and Discussion

The evaluation methodology in the experiments was a 5-fold cross-validation, where the 2262 examples were divided into five groups or folds to train, validate, and test the system with different data. Specifically, 80% of the data was used for training, 10% for validation, and 10% for testing. The data splitting and the system evaluation were repeated five times, with different subsets. The final results were the average of the outcomes obtained from all iterations.

The validation subset is used to optimize the hyperparameters of the transformer-based architecture:

- Optimizer: Adam optimizer
- Number of epochs: 250
- Batch size: 32
- Number of attention heads: 6
- Learning rate: 0.001
- Dropout rate: 0.3
- Intermediate dimension: 1024
- Embedding dimension: 128

In the next sections, we evaluate in detail the process of generating the landmarks and the detection of the stop frame. These two aspects were independently examined to analyze which is the optimal setup for both tasks (landmark generation and stop detection) in terms of padding strategy, interpolation, and the use of data augmentation.

### 5.1. Motion Generation

To assess the quality of motion generation, we used the dynamic time warping (DTW) distance that compares ground truth and generated motion sequences. The goal of this metric is to find the best mapping between the predicted sequence of landmarks and the ground truth sequence. This best mapping corresponds to the minimum accumulated distance, computed by the DTW using dynamic programming. This distance is divided by the length of the sign (in frames). The required distance between two landmark frames is defined by Equation (1), where the subscripts “O” and “P” indicate the original and predicted frames, respectively, and the subscript “i” denotes the landmark number. In this work, we used the DTW functionality obtained from the dtaidistance.dtw_ndim.distance Python function [31].

(1) $$DTW\left(O,P\right)=\sum_{i=0}^{N}\sqrt{{\left(x_{Oi}-x_{Pi}\right)}^{2}+{\left(y_{Oi}-y_{Pi}\right)}^{2}}$$

It is important to highlight that, in this subsection, we compute the DTW distance per frame between the predicted landmark-based motion sequence and the ground truth, assuming a perfect detection of the stop frame. As needed by the analysis, we will include the effect of automatically detecting the stop frame.

**Figure 10:**
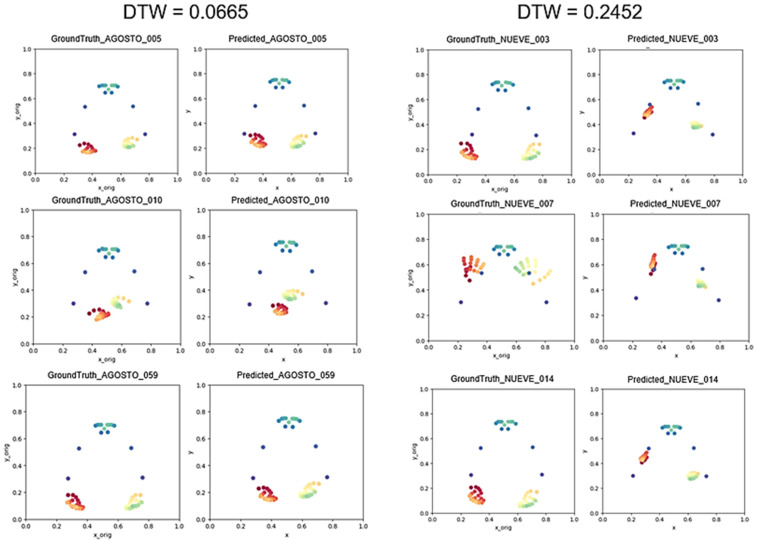
Examples of DTW values and the corresponding frame sequences (every landmark has a different color).

Figure 10 shows two DTW values obtained for two frame sequences to provide a visual reference of this performance metric. The left part shows a low DTW value for a well-predicted sign and the right part shows a very high value for a badly generated sign (big differences between predicted and ground truth frames).

In the next subsection, we compare the different padding strategies, interpolation approaches, and data augmentation techniques.

#### 5.1.1. Evaluation of Padding Strategies

**Figure 11:**
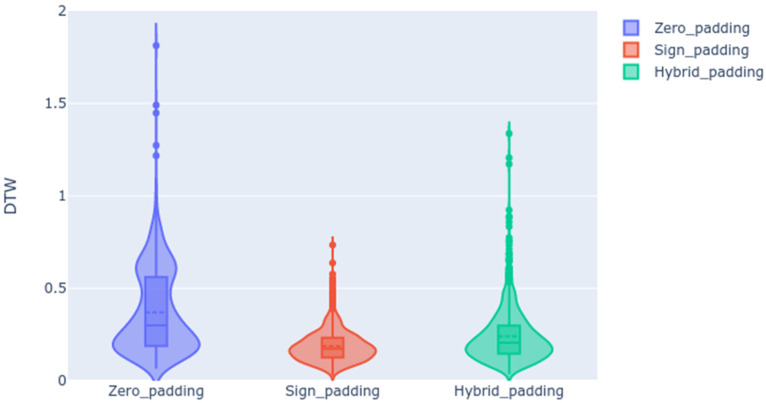
DTW results for the validation subset considering the different padding strategies.

Figure 11 shows the DTW distance distributions along the testing subset using violin charts. As shown, the zero padding strategy, where we included zeros after the sign, provided higher values of DTW distance between the predicted landmark-based motion sequence and the ground truth. However, sign padding and hybrid padding obtained better distributions of DTW values (lower DTW distances). Comparing these last two strategies, we can conclude that the best one is sign padding because of its lower DTW value. The violin charts efficiently illustrate the distributions of the DTW distances for all predicted examples. This representation allows us to compare different distributions obtained in different experiments. These DTW distributions provide more information than considering only the DTW average in each experiment. For example, we observed different patterns when comparing the padding strategies: using zero padding, the DTW values were distributed in two distinct Gaussian distributions, while when employing hybrid padding, the DTW values were spread across a broader Gaussian shape, punctuated by a few outliers. In contrast, DTW distances when employing sign padding were concentrated within a narrower Gaussian, with lower DTW values compared to the two previous strategies.

Additionally, Table 1 includes the average DTW values and the standard deviation for each analyzed scenario.

We hypothesize that sign padding works better than the other two strategies because we provide the transformers with more examples and opportunities to learn how to generate the landmarks of the next frame.

#### 5.1.2. Evaluation of Interpolation Strategies

Having concluded that the best padding strategy for landmark generation is sign padding, this section will examine the effect of interpolation. As commented previously, the purpose of introducing intermediate frames is to smooth the landmark sequences and facilitate the learning process of the transformer-based architecture.

**Figure 12:**
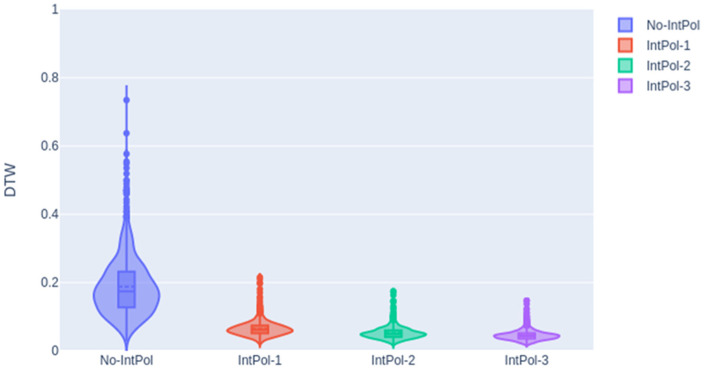
DTW results for the validation subset considering the different interpolation strategies using the sign padding strategy.

Figure 12 illustrates the distributions obtained for the DTW distance when adding one interpolated frame (“IntPol-1”), two interpolated frames (“IntPol-2”), and finally, three interpolated frames (“IntPol-3”). In this figure, narrower Gaussian distributions were obtained when interpolations were computed compared to the no interpolation experiment.

Table 2 presents the average DTW values and standard deviation for each interpolation.

From these results, we can conclude that the interpolation helps the training process obtain a significant reduction in the DTW distance: the average distance is around 0.05, and all examples are lower than 0.2. As shown, when increasing the number of interpolated frames, we observed a saturation behavior of the system with a small difference between two and three interpolations. We think that using only two interpolated frames can be a good compromise between performance and time processing.

#### 5.1.3. Data Augmentation

**Figure 13:**
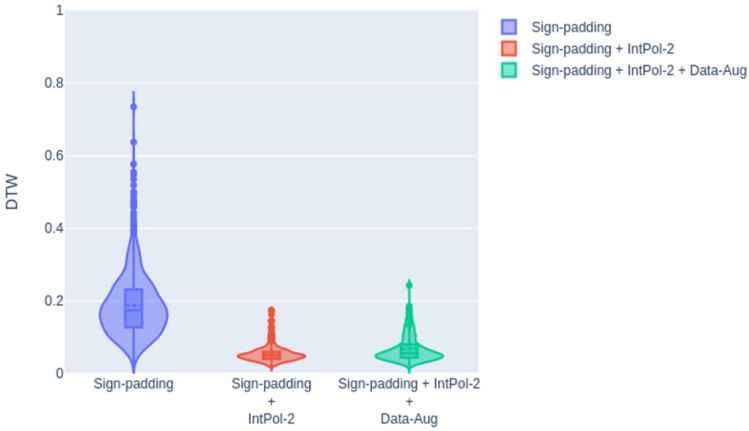
DTW results for the validation subset including the different techniques.

Finally, we evaluated the system incorporating synthetic data in the training of the transformer. In the next figure (Figure 13), we show the DTW distance distribution for the original sign padding strategy (without interpolation or data augmentation), sign padding with interpolation (without data augmentation), and sign padding with interpolation and data augmentation. As shown in this figure, similar Gaussian distributions were obtained when including the data augmentation compared to using only the interpolation.

Table 3 shows the average DTW values and the standard deviations.

As shown, we did not obtain any improvement when introducing the augmented data during the training of the transformer. Analyzing the results, we observed a good match between the training and testing subsets (all examples have the same point of view), and introducing modified data did not improve the results. In future work, we will consider more different subsets for training and testing.

### 5.2. Stop Detection Evaluation

To evaluate the stop detection module, since this detection is a binary classification task, standard evaluation metrics for two classes were used for evaluating the performance: accuracy, receiver operating characteristic (ROC) curves, and area under the curve (AUC). Accuracy is defined as the ratio between the number of correctly classified samples and the number of total samples. ROC is a curve representing the true positive rate (TPR) versus the false positive rate (FPR) obtained at several thresholds settings. AUC refers to the ratio of the area under the ROC curve. This measure represents the separability between two classes. The higher the AUC, the better the model distinguishes between the two classes of the binary classification problem.

For accuracy values, we also considered confidence intervals [32] to analyze the statistical significance of the differences between the two experiments. We can establish a significant distinction between the results of two experiments when their confidence intervals do not overlap. Equation (2) represents the computation of confidence intervals attached to a specific metric value and *N* samples when the confidence level is 95%.

(2) $$CI\left(95\%\right)=\pm 1.96\sqrt{\frac{accuracy\times 100-accuracy)}{N}}$$

To analyze the statistical significance of the AUC differences in the two experiments, we provided two-tailed *p*-values.

As in the previous section, we analyzed the impact of the different padding strategies, interpolation approaches, and data augmentation techniques for the stop detection. 

#### 5.2.1. Evaluation of Padding Strategies

**Figure 14:**
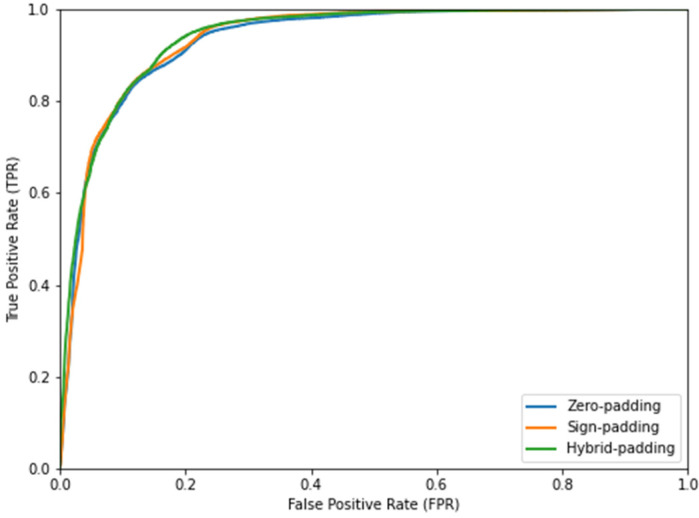
ROC curves for the stop output detection depending on the padding strategy.

To determine the best padding strategy, we analyzed the AUC values with the corresponding *p*-values. Table 4 presents the AUC values for each padding strategy, along with the study of the two-tailed *p*-value between the sign padding and hybrid padding strategies, as they are the best strategies. Figure 14 shows the ROC curves for the three padding strategies.

As observed, all padding strategies showed very good results with AUCs over 0.93. The hybrid padding strategy provided the highest AUC value. Furthermore, the *p*-value analysis confirmed the significance of the difference between hybrid padding and the rest of strategies.

#### 5.2.2. Evaluation of Interpolation Strategies

**Figure 15:**
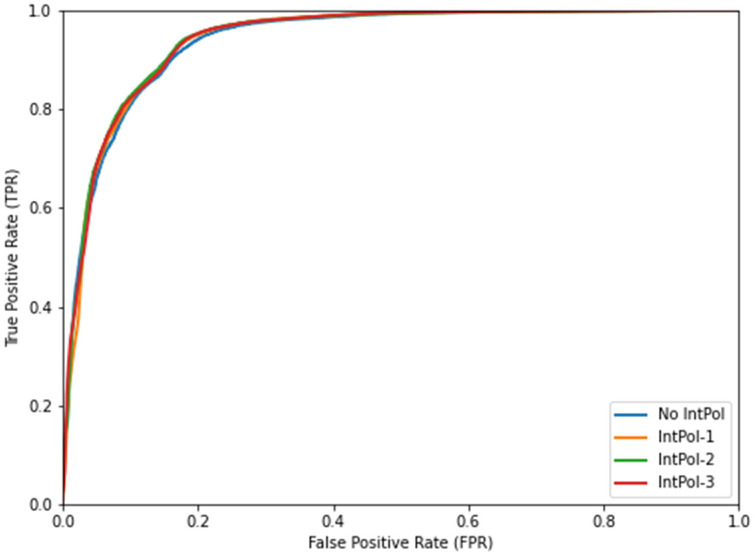
ROC curves for stop detection depending on the interpolation approach using the hybrid padding strategy.

After selecting the hybrid padding strategy, we proceeded to analyze the effect of interpolation on stop detection. Similarly to the previous section, we analyzed AUC values and ROC. Table 5 shows the AUC values and *p*-value analysis for the three interpolation approaches using a hybrid padding strategy, and Figure 15 shows the associated ROC curves. As observed in Table 5, the *p*-value between the AUC of “No Inter” and “IntPol-1” experiments, and the *p*-value between the AUC of “IntPol-2” and “IntPol-3” were higher than 0.001, which implies that the differences between these experiments were not statistically significant. However, the *p*-value between the AUC of “IntPol-1” and “IntPol-2” was lower than 0.001, which determines a significant improvement: including two interpolated frames provided significant better results than using no interpolation.

As observed, introducing interpolated frames obtained improvements compared to no interpolation. These results are consistent with the improvement already obtained in landmark generation. When comparing the number of interpolated frames, we observed a better AUC with two interpolated frames, but the differences were not significant. We can conclude that interpolating with two frames is a good compromise between performance and processing time, and it is also consistent with the best scenario regarding interpolation obtained in landmark generation. 

#### 5.2.3. Evaluation of Data Augmentation

Lastly, similar to the evaluation of motion generation, we assessed the effect of applying data augmentation by employing the hybrid padding strategy and adding two rows of interpolation. This analysis will help us to understand the impact of data augmentation on the overall performance.

Table 6 shows the AUC values and the *p*-value between the hybrid padding + interpolation of two frames strategy and the hybrid padding + interpolation of two frames + data augmentation strategy.

As observed, applying data augmentation resulted in a better AUC value for the stop detector due to the increase in the number of examples in the training process. Additionally, the analysis of the *p*-value confirmed statistical independence between the results. This significant difference supports the conclusion that the interpolation and data augmentation strategies improved the performance of the stop detector. 

Despite obtaining better results with data augmentation in the stop detector, subsequent experiments continued without this strategy. This decision was based on the observation that data augmentation did not provide any improvement in landmark generation. Thus, to maintain consistency and focus on the most effective strategies, data augmentation was not applied to subsequent experiments for landmark generation.

#### 5.2.4. Evaluation of the Decision Threshold

In order to extend the analysis, we showed the detection accuracy depending on the decision threshold. We also showed the average length (in number of original frames) of the ground truth and predicted sequences, and the length difference between them. We carried out this analysis with the architecture obtained in the previous subsections (hybrid padding with two interpolated frames). Table 7 shows the different values of validation accuracy for different thresholds, along with their confidence intervals. 

The table also includes the difference between the number of predicted frames by the transformer and the number of frames of the ground truth. This analysis also allowed us to examine the effect of the threshold on this aspect.

When increasing the threshold, the average number of predicted frames increases. Considering that the automatic system can generate signs with a different pace compared to the ground truth (faster or slower sign representations), we decided to select the threshold based on the detection accuracy. In this case, we can conclude that the threshold reporting the best validation accuracy was 0.5.

**Figure 16:**
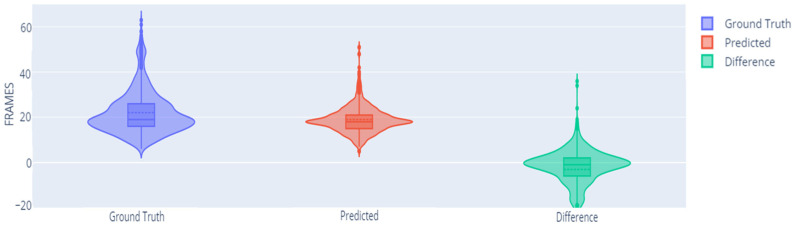
Number of predicted and reference frames.

In Figure 16, a violin plot is displayed representing the predicted frames, the reference frames, and their differences for a 0.5 threshold. It can be observed that while there are some signs with slight differences, many signs have a similar number of frames.

## 6. Final Experiment

In this section, we present the results with the test subset combining motion generation and stop detection (with a 0.5 threshold) in the same system. For this purpose, we considered a scenario where both systems performed well, and we chose the sign padding strategy, as it was the best scenario for landmark generation, along with two interpolated frames. Table 8 displays the DTW value obtained along with its standard deviation and the comparison between the analysis made in the previous section. 

**Figure 17:**
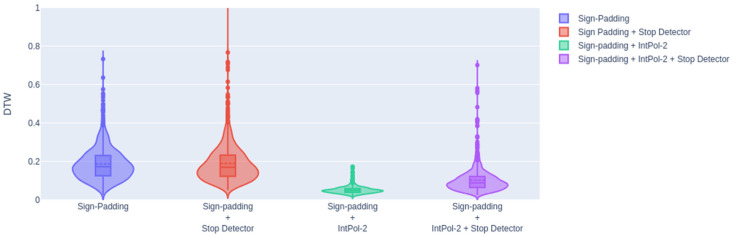
DTWs results comparing best scenarios with the final experiment.

Figure 17 displays a violin plot representing these values along with their distributions, where we observed that when including the stop detector, the distributions of DTW values were wider comparing to the ones when it was not included. 

As observed, when adding the stop detector, the DTW worsened compared to the best scenario analyzed in the previous sections. However, even with the addition of the stop detector, we achieved a DTW value lower than the one obtained in the scenario where only the sign padding strategy was applied without the stop detector (using perfect sign limits). The degradation when using the stop detector was not constant in all experiments; for example, when considering the stop detector without interpolation, we observed a higher performance degradation (average DTW of 0.1908 ± 0.0992) than in the case with interpolation (average DTW of 0.1057 ± 0.0659). This result is because both systems (landmark generation and stop detector) share the same transformer (a relevant part of the deep learning architecture), and the quality of the transformer has a strong influence on the performance of both tasks.

Considering previous research focusing on generating pose animation from natural language, a previous work [12] provided an average positional error of 49.5 mm. In our system, the landmarks are scaled within the range of 0 to 1, and the frames represent an individual from the hip upward, equating to approximately 1.2 m. Through our analysis, we determined an accumulated error of 0.1 across the 57 landmarks (for our best system). This error corresponds to an average error of 2.1 mm per landmark. This result implies a noticeable improvement comparing to the previous work. Moreover, certain landmarks, such as those related to the face and shoulders, remain relatively static (very low errors), whereas others, like those included in arms and hands, exhibit higher errors. Nevertheless, the error in these points is lower than 10 mm.

## 7. Conclusions

This work proposed a sign language motion generation system using sign characteristics or phonemes as inputs. The sign characteristics are represented using HamNoSys, a notation system for any sign language developed at the University of Hamburg. The sign phonemes provide information about sign characteristics like hand configuration, localization, or movements. This information is necessary to generate sign motion with a high level of detail. The proposed architecture based on the transformer also includes a stop detection module for predicting the end of the generation process. Both aspects, motion generation and stop detection, were evaluated in detail considering specific performance metrics. 

In order to improve the proposed architecture, different padding strategies, interpolation approaches, and data augmentation techniques were considered and evaluated. Regarding the padding strategies, the repetition of the sign (instead of including zeros) empowered the learning process of the transformer reporting significant performance improvements. The introduction of interpolated frames smoothed the frame sequence, facilitating the learning process of the transformer and reporting major improvements. This was the most significant contribution. The introduction of data augmentation in the training did not provide significant improvements due to the fact that the data used for training and testing are very similar. More difficult scenarios must be addressed to demonstrate its utility.

Regarding the limitations of the work, the motion generation is limited to sign-level representation, instead of complete sentences in sign languages. In future works, we will explore the idea of generating motion from complete sentences instead of from individual signs. Additionally, we would like to reproduce the experiments with bigger datasets. Finally, we could incorporate an additional motion evaluation metric based on a deep learning classifier. The idea is to develop a sign recognition system and evaluate how well this system can recognize the generated signs.

## Figures and Tables

**Table 1:** DTW mean and standard deviation depending on the padding strategy.

	Zero Padding	Sign Padding	Hybrid Padding
DTW mean and std	0.3738 ± 0.2269	0.1880 ± 0.0857	0.2406 ± 0.1423

**Table 2:** DTW mean and standard deviation depending on the interpolation approach.

	No IntPol	IntPol-1	IntPol-2	IntPol-3
DTW mean and std	0.1880 ± 0.0857	0.0640 ± 0.0215	0.0523 ± 0.0177	0.0456 ± 0.0149

**Table 3:** DTW mean and standard deviation considering data augmentation.

	Sign Padding	Sign Padding + IntPol-2	Sign Padding + IntPol-2 + Data Augmentation
DTW mean and std	0.1880 ± 0.0857	0.0523 ± 0.0177	0.0677 ± 0.0340

**Table 4:** AUC. Padding strategies.

	Zero Padding		Sign Padding		Hybrid Padding	
AUC	0.93282		0.93571		0.94025	
*p*-value	0.013971			0.00007		

**Table 5:** AUC. Interpolation.

	No Inter	IntPol-1	IntPol-2	IntPol-3
AUC	0.94025	0.94133	0.94379	0.94297
*p*-value	0.264679			
		0.000526		
			0.161873	

**Table 6:** AUC. Data Augmentation.

	Hybrid Padding	Hybrid Padding + IntPol-2	Hybrid Padding + IntPol-2 + Data Augmentation
AUC	0.94025	0.94379	0.97653
*p*-value	0.000097		
		<0.000001	

**Table 7:** Threshold analysis for the stop output detection: validation accuracy, confidence interval, average number of predicted frames, average number of frames in the reference, and average difference (best results in bold font).

Threshold	0.1	0.2	0.3	0.4	0.5	0.6	0.7	0.8	0.9
Val. Accuracy	89.06	89.89	90.28	90.49	**90.64**	89.37	86.50	85.49	83.85
Confidence Interval	±0.218	±0.210	±0.207	±0.205	**±0.203**	±0.215	±0.238	±0.250	±0.257
Average # Pred.	15.03	16.08	16.99	18.53	**19.23**	21.17	25.32	26.99	29.37
Average # Refer.	21.60	21.60	21.60	21.60	**21.60**	21.60	21.60	21.60	21.60
Average Difference	−6.35	−5.52	−4.61	−3.07	**−2.37**	−0.43	3.72	5.39	7.77

**Table 8:** DTW mean and standard deviation including the stop detector.

	Sign Padding	Sign Padding + Stop Detector (0.5)	Sign Padding + IntPol-2	Sign Padding + IntPol-2 + Stop Detector (0.5)
DTW mean and std	0.1880 ± 0.0857	0.1908 ± 0.0992	0.0523 ± 0.0177	0.1057 ± 0.0659

## Data Availability

The dataset can be obtained by requesting it via email to the corresponding author.
